# Fulminant hepatic and multiple organ failure following acute viral tonsillitis: a case report

**DOI:** 10.1186/s13256-015-0777-3

**Published:** 2016-01-20

**Authors:** Ursina Bechtel-Grosch, Charles Beguelin, Sabina Berezowska, Jean-Francois Dufour, Jukka Takala, Joerg C. Schefold

**Affiliations:** 1 Department of Intensive Care Medicine, Inselspital, Bern University Hospital, CH 3010 Bern, Switzerland; 2 Department of Infectious Diseases, Inselspital, Bern University Hospital, CH 3010 Bern, Switzerland; 3 Institute of Pathology, University of Bern, CH 3010 Bern, Switzerland; 4 Department of Visceral Surgery and Medicine, Inselspital, Bern University Hospital, CH 3010 Bern, Switzerland

**Keywords:** Critical illness, Hepatitis, MOF, Sepsis, Septic shock, Viral infection

## Abstract

**Background:**

Pyogenic tonsillitis may often be observed in the general Western population. In severe cases, it may require antibiotic treatment or even hospitalization and often a prompt clinical response will be noted. Here we present an unusual case of progressive multiple organ failure including fulminant liver failure following acute tonsillitis initially mistaken for “classic” pyogenic (that is bacterial) tonsillitis.

**Case presentation:**

A 68-year-old previously healthy white man was referred with suspicion of pyogenic angina. After tonsillectomy, he developed acute liver failure and consecutive multiple organ failure including acute hemodynamic, pulmonary and dialysis-dependent renal failure. Immunohistopathological analysis of his tonsils and liver as well as serum polymerase chain reaction analyses revealed herpes simplex virus-2 to be the causative pathogen. Treatment included high-dose acyclovir and multiorgan supportive intensive care therapy. His final outcome was favorable.

**Conclusions:**

Fulminant herpes simplex virus-2-induced multiple organ failure is rarely observed in the Western hemisphere and should be considered a potential diagnosis in patients with tonsillitis and multiple organ failure including acute liver failure. From a clinical perspective, it seems important to note that fulminant herpes simplex virus-2 infection may masquerade as “routine” bacterial severe sepsis/septic shock. This persevering condition should be diagnosed early and treated goal-oriented in order to gain control of this life-threatening condition.

## Background

In the general Western population, pyogenic (that is bacterial) tonsillitis may often be observed and it is known to account for a large number of pre-clinical consultations. In severe cases, it may require antibiotic treatment or even hospitalization and a prompt clinical response will often be noted [1–3]. Here we present an unusual case of progressive multiple organ failure including fulminant liver failure following acute non-bacterial tonsillitis. We will furthermore report and discuss pitfalls in the clinical management of such life-threatening non-bacterial tonsillitis.

## Case presentation

A 68-year-old white man with an insignificant past medical history (including sexual history) was referred to our tertiary care academic center with symptoms of acute tonsillitis, clinically not responding to antibiotic treatment with oral amoxicillin-clavulanic acid. Following a 2-day course of moderate fever (38 °C) and sore throat with highly elevated infection parameters of C-reactive protein 280 mg/l, white blood cell count (WBC) 4.8 G/L, and platelets 106 G/L, he was hospitalized for intravenous antibiotic treatement. An initial physical examination at admission was unremarkable. Due to a progressive elevation in his inflammatory markers along with clinical deterioration and progressive tonsillar inflammation, a left-sided tonsillectomy was performed 48 hours after his hospital admission. During his postoperative course, fulminant acute liver failure (ALF) was observed and he was transferred to our intensive care unit (ICU) for further investigations and supportive intensive care treatment.

**Fig. 1:**
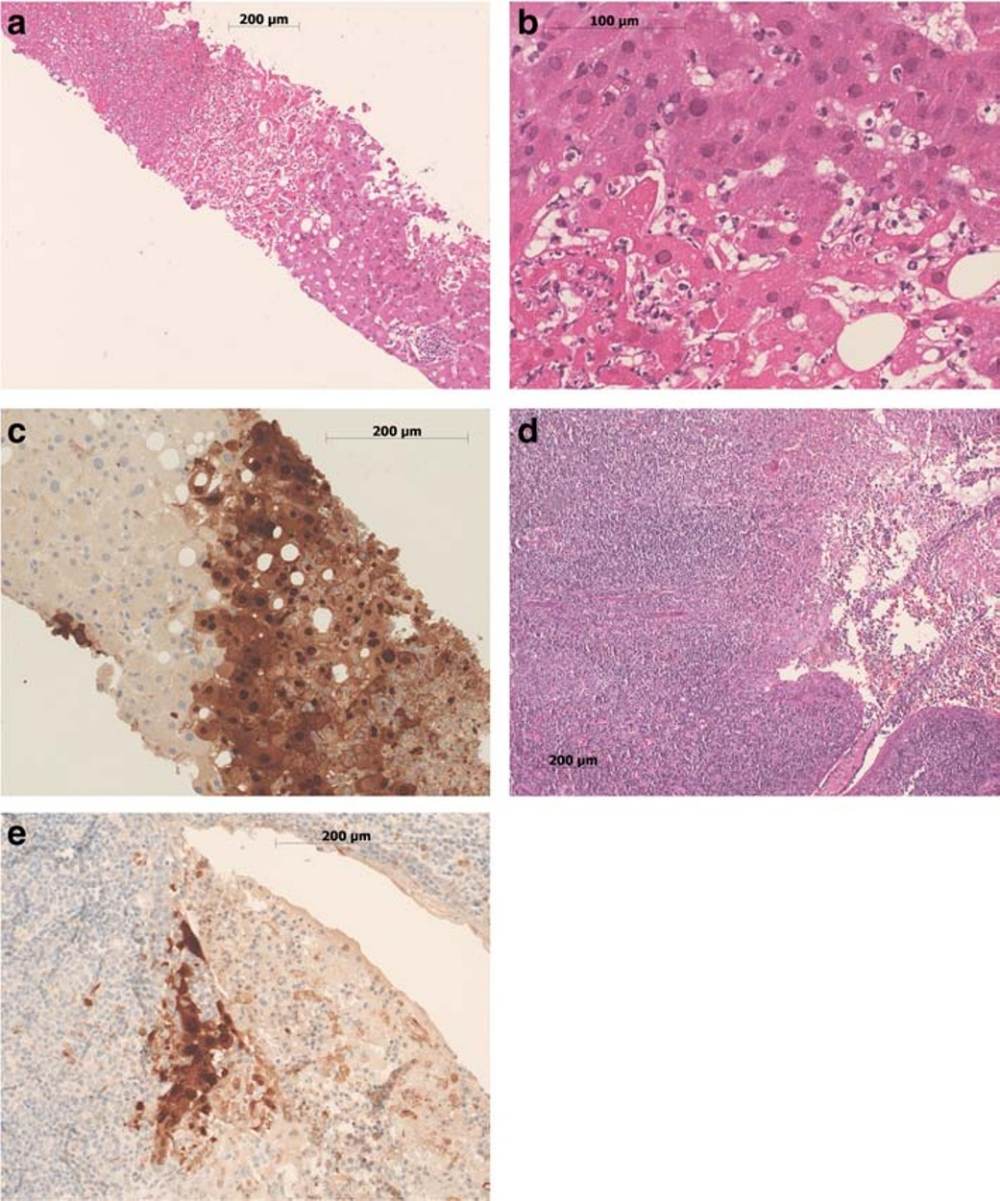
Hepatic (**a-c**) and tonsillar (**d-e**) histology. Liver biopsy tissue shows patchy necrosis comprising 20 to 30 % of the submitted tissue (**a** hematoxylin and eosin ×100), with nuclear viral inclusions (**b** hematoxylin and eosin ×400), immunohistochemically positive for herpes simplex virus-2 antigen (**c** ×400). Tonsillar tissue examination discloses acute ulcerating tonsillitis (**d** hematoxylin and eosin ×100) with patchy positivity for herpes simplex virus-2 antigen in some remnants of surface epithelium adjacent to the ulcer and in necrotic tissue overlying the ulcer (**e** ×200). Photomicrographs were taken on a Zeiss Axiophot2 microscope

At ICU admission, laboratory analyses revealed leukopenia (1.8 G/l) with pan-lymphopenia and marked elevation of indices of liver dysfunction (Table 1). Toxic, vascular/ischemic, autoimmune, or metabolic liver disease was considered unlikely following anamnesis and respective laboratory tests (please also refer to Table 1). In addition to laboratory signs of ALF, the patient soon developed acute kidney injury/failure: risk, injury, failure, loss of kidney function, and end-stage kidney disease (RIFLE) category “F”. An emergency abdominal ultrasound revealed moderate hepatic steatosis, and computed tomography imaging revealed inhomogeneous hepatic steatosis, ascites and moderate splenomegaly. A retropharyngeal abscess was ruled out. Infective tonsillitis-induced septic shock with progressive multiple organ failure was suspected and the initial antibiotic treatment using amoxicillin-clavulanic acid was changed to ciprofloxacin, Cubicin (daptomycin) and metronidazole. During the further course of the disease, supportive medical treatment included high-dose acetylcysteine, vitamin K and lactulose. However, further worsening of liver function tests was noted and a decision for a transjugular liver biopsy was established 48 hours following his ICU admission. Initial histologic findings revealed diffuse hepatic necrosis compatible with viral hepatitis (Fig. 1); empirical antiviral treatment using ganciclovir (500 mg single-shot, followed by 450 mg twice a day) was commenced in order to cover cytomegalovirus (CMV) and herpes simplex virus (HSV). In addition, initial results showed seropositivity for HSV-2 immunoglobulin G (IgG), Epstein–Barr virus (EBV) IgG and CMV IgG. Serological testing for human immunodeficiency virus (HIV) and for hepatitis B and hepatitis C revealed negative results. Final histological results of his liver then demonstrated significant HSV-1 and HSV-2 immunopositivity. In combination with strongly elevated HSV-2 serum polymerase chain reaction (PCR) levels (10^6^ to 10^7^ HSV-2 copies/ml) a formal diagnosis of HSV-2-positive hepatitis was established and treatment with ganciclovir was changed to acyclovir. The antibiotic therapy was stopped at this point in time. Due to strong HSV-positivity of hepatic tissue, tonsillar histologies were re-evaluated for HSV and marked respective immunopositivity was noted (Fig. 1). Thus, clinical and histological data strongly suggested primary HSV-2 tonsillitis.

**Table 1:** Paraclinical presentation at intensive care unit admission. Reference ranges are indicated

C-reactive protein	<5	mg/L	393
Creatinine	59–104	μmol/L	158
Aspartate aminotransferase	<50	U/L	3829
Alanine aminotransferase	<50	U/L	2310
Gamma-glutamyltransferase	<60	U/L	148
Ammonia	15–55	μmol/L	94
Bilirubin, total	<17	μmol/L	101
Bilirubin direct	<5	μmol/L	97
Glucose	4.56–6.38	mmol/L	8.4
Prothrombin time	70–130	%	64
Factor V	78–153	%	45
Hemoglobin	135–168	g/L	134
Thrombocytes	140–380	G/L	53
Leukocytes	3.5–10.5	G/L	1.8
Neutrophils	1.60–7.40	G/L	1.66
Eosinophils	0.02–0.40	G/L	0.01
Basophils	0.00–0.15	G/L	0
Monocytes	0.20–0.93	G/L	0.02
Lymphocytes	1.10–3.50	G/L	0.09
Neutrophil bands	3–18	%	63.5
Segmented neutrophils	35–67	%	29

Along with the development of fulminant ALF, the major clinical concern was progressive multiple organ failure. Despite initial “rescue”-steroid dosing, high doses of norepinephrine (peak dose of 0.3 μg/kg/minute) were required and progressive capillary leak was observed. Following a consecutive 48-hour trial of noninvasive ventilation, the patient required invasive mechanical ventilation as a consequence of progressive muscular exhaustion. He subsequently developed anuric acute renal failure and renal replacement therapy with intermittent hemodialysis was initiated. Due to deranged coagulation in which his platelet count was 56 G/l, prothrombin time was 38 %, and international normalized ratio (INR) was 1.82, with recurrent bleeding at the tonsillectomy site and progressive disseminated intravascular coagulation, an operative revision with ligation of tonsillar arteries was deemed necessary. High urgency liver transplantation was discussed as a therapeutic option but was refrained from due to persisting uncontrolled infection/multiple organ failure with an expected badverse outcome.

**Fig. 2:**
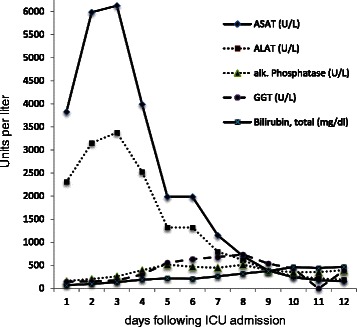
Course of key laboratory parameters used to assess cellular damage and hepatic dysfunction in patient with multiple organ failure. Laboratory indices at respective days following intensive care unit admission. *ALAT* alanine aminotransferase, *alk*. alkaline, *ASAT* aspartate aminotransferase, *GGT* gamma-glutamyltransferase

During his course at the ICU, repeated drainage of ascites was necessary. Aspartate aminotransferase (ASAT) and alanine aminotransferase (ALAT) serum levels peaked at day 3 (ASAT 6126U/l, ALAT 3377U/l), and elevated serum ammonia (123 μmol/l), alkaline phosphatase levels (520 U/l), and total bilirubin levels were noted (Fig. 2). His serum factor V levels were 24 % at their lowest (following prior infusion of fresh frozen plasma). Continuous norepinephrine application was required for the ensuing 7 weeks due to severe vasoplegic shock and capillary leak. Extensive sepsis-induced generalized edema was noted and cardiogenic failure was ruled out by repeated echocardiography. As weaning from mechanical ventilation was complicated by capillary leakage and consecutive volume overload, a dilative tracheostomy was performed at day 12. In the ensuing days, anuria persisted and continuous veno-venous hemodiafiltration was performed. Repeated cultures of urine, blood, sputum and stool specimens all revealed insignificant microbiological results.

**Fig. 3:**
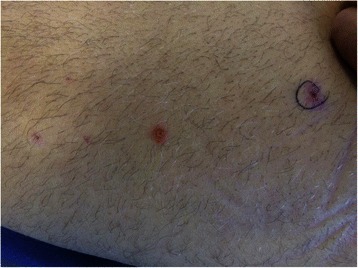
Eruptions demonstrating generalized herpetic rash at day 8 following intensive care unit admission (left thigh)

Over the ensuing days, a repeated generalized rash with herpetic vesicles under continued high-dose acyclovir treatment (initial dose of 2700 mg/day followed by 1500 mg/day for approximately 30 days with respective adjustment to renal function) was noted (Fig. 3). In the light of a persevering course of HSV-2-induced multiple organ failure and continued high titers of “viral load” (that is 10^5^ to 10^6^ copies of HSV-2 per ml at days 14 and 21), geno-typization of the HSV-2 virus for acyclovir resistance was performed which revealed negative results. The patients’ condition finally stabilized after 33 days of treatment. However, due to persisting lymphopenia and in view of the overall clinical severity, a prophylactic treatment with acyclovir (400 mg defined daily dose) was continued.

After approximately 1 month of intensive care treatment, an overall stabilization of his condition was noted. Norepinephrine was tapered and weaning from the ventilator was advanced. A diagnosis of ICU-acquired weakness was established and rehabilitative measures were initiated.

## Discussion

In the light of the available literature, HSV-2-induced multiple organ failure is a rare condition. HSV infection in adults is commonly associated with cutaneous facial (HSV-1) or urogenital infections (HSV-2) [4]. Of interest, approximately 60 % of the Western population is HSV-1 colonized, whereas HSV-2 colonization occurs in approximately 12 % of cases [4]. Oral HSV infection in adults may present as severe pharyngitis accounting for approximately 6 % of cases in young adults. However, HSV-2 seems even scarcer and accounts for approximately <0.5 % of cases [5]. Systemic HSV infection with visceral viral dissemination may often present as ALF due to fulminant hepatic necrosis but represents less than 1 % of cases of ALF [6]. Due to a lack of specific therapeutic options, HSV hepatitis is associated with mortality rates of up to 90 % [7]. High-risk populations for a fatal course are immunosuppressed patients and pregnant patients (third trimester). Of interest, immunocompetency was deemed in approximately 24 % of severely affected patients [7]. In our patient, testing for chronic viral disease including HIV testing, standard examinations including WBCs (following recovery from lymphopenia), immunoglobulins, distribution of immune cell subpopulations, and assessment of major histocompatibility complex (MHC) class II expression human leukocyte antigen- DR (HLA-DR) [8, 9] did not reveal a major immunocompromising condition. Nevertheless, in a case such as in our patient, a potential underlying immune deficiency should be ruled out.

Because of an unspecific clinical presentation, a diagnosis of HSV-induced hepatitis may frequently be missed and data demonstrate that the majority (that is 58 %) of patients are diagnosed post-mortem [7]. Improved survival rates are reported following early initiation of antiviral treatment. Retrospective analyses of patients with HSV hepatitis demonstrate significantly lower mortality rates following early acyclovir treatment when compared to controls (51 % versus 88 %). Treatment delay is associated with higher need for liver transplantation and higher mortality rates [7]. Thus, early initiation of antiviral therapy may be regarded mandatory to prevent fatal outcomes. Orthotopic liver transplantation (OLT) in the context of disseminated HSV infection should be evaluated carefully, as the risk for HSV recurrence seems increased and lifelong treatment with acyclovir may be needed following OLT [10]. Although there is a lack of conclusive data, intensivists should consider treating this life-threatening condition with antiviral medication over a sustained period of time until profound clinical improvement is observed. However, optimal dosage, especially in the presence of acute renal failure, and optimal timing of treatment for HSV-2-induced multiple organ failure remains elusive. In addition, it should be kept in mind that serum PCR levels (that is, respective assessment of viral load) may reveal positive results for a sustained period in time. This may be due to amplification of non-live viral particles and may be misleading in this clinical scenario.

## Conclusions

In conclusion, this case demonstrates that HSV-2-induced multiple organ failure may develop in immunocompetent hosts following HSV-2 infectious tonsillitis. The clinical picture may masquerade as “routine” bacterial septic shock induced by bacterial tonsillitis and may lead to fatal clinical decisions if not recognized early and treated accordingly.

## Consent

Written informed consent was obtained from the patient for publication of this case report and accompanying images. A copy of the written consent is available for review by the Editor-in-Chief of this journal.
